# PRDM16 Regulating Adipocyte Transformation and Thermogenesis: A Promising Therapeutic Target for Obesity and Diabetes

**DOI:** 10.3389/fphar.2022.870250

**Published:** 2022-04-08

**Authors:** Na Jiang, Ming Yang, Yachun Han, Hao Zhao, Lin Sun

**Affiliations:** Hunan Key Laboratory of Kidney Disease and Blood Purification, Department of Nephrology, The Second Xiangya Hospital of Central South University, Changsha, China

**Keywords:** positive regulatory domain zinc finger region protein 16 (PRDM16), diabetes, obesity, adipocyte, browning

## Abstract

Given that obesity and diabetes have been major public health concerns and that disease morbidities have been rising continuously, effective treatment for these diseases is urgently needed. Because adipose tissue metabolism is involved in the progression of obesity and diabetes, it might be efficient to target adipocyte metabolic pathways. Positive regulatory domain zinc finger region protein 16 (PRDM16), a transcription factor that is highly expressed in adipocytes, plays a key role in adipose tissue metabolism, such as the browning and thermogenesis of adipocytes, the beigeing of adipocytes, the adipogenic differentiation of myoblasts, and the conversion of visceral adipocytes to subcutaneous adipocytes. Furthermore, clinical and basic studies have shown that the expression of PRDM16 is associated with obesity and diabetes and that PRDM16 signaling participates in the treatment of the two diseases. For example, metformin promotes thermogenesis and alleviates obesity by activating the AMPK/αKG/PRDM16 signaling pathway; rosiglitazone alleviates obesity under the synergistic effect of PRDM16; resveratrol plays an antiobesity role by inducing the expression of PRDM16; liraglupeptide improves insulin resistance by inducing the expression of PRDM16; and mulberry leaves play an anti-inflammatory and antidiabetes role by activating the expression of brown fat cell marker genes (including PRDM16). In this review, we summarize the evidence of PRDM16 involvement in the progression of obesity and diabetes and that PRDM16 may be a promising therapy for obesity and diabetes.

## Introduction

Diabetes is a serious disease with a wide range and severe impact that endangers human health worldwide, and its incidence has been increasing continuously for the past 50 years (Aschner et al., 2021). By 2019, the prevalence of diabetes reached 9.3% and will increase up to 10.2% by 2030 and 10.9% by 2045 (Saeedi et al., 2019). In recent years, obesity has also become a major public health concern, increasing the risk of chronic diseases such as hypertension, cardiovascular disease, and diabetes (Gadde et al., 2018). Obesity is related not only to the development of diabetes but also to the development of diabetes-related complications (Piché et al., 2020). Obesity and diabetes are closely related, and the mechanisms involved are intricate (Saxton et al., 2019). A review of the literature shows that adipose tissue metabolism plays an important role in diabetes and obesity and is likely an important bridge between them (Kaisanlahti and Glumoff, 2019; Saxton et al., 2019). Adipose tissue is divided into three types: white fat, brown fat, and beige fat. White fat is mainly responsible for energy storage, while brown fat and beige fat are mainly responsible for energy release (Zwick et al., 2018). A number of studies have shown that the browning of adipose tissue and the thermogenesis of brown fat affect insulin resistance and glucose metabolism, which are of great significance for the improvement of obesity and diabetes (Baskaran et al., 2016; Hasegawa et al., 2018; Raffaele et al., 2020). Emerging evidence shows that positive regulatory domain zinc finger region protein 16 (PRDM16), as a transcription factor (Seale et al., 2007), can participate in adipocyte transformation and thermogenesis through various pathways, such as the maintenance of brown adipose tissue morphology and thermogenesis (Seale et al., 2011; Chi and Lin, 2018; Gan et al., 2018), adipogenic differentiation of myoblasts (Frühbeck et al., 2009; Borensztein et al., 2012; Jiang et al., 2018), the conversion of visceral adipocytes to epidermal adipocytes (Ding et al., 2016; Liu L. et al., 2021a), and the beigeing of white adipocytes (Cohen et al., 2014; Wang et al., 2019). It is speculated that PRDM16 protein could be an effective way to treat obesity and diabetes. In this review, we summarized the role of PRDM16 in adipose tissue and proposed the use of PRDM16 as a potential target for the treatment of obesity and diabetes.

## PRDM16

### Structure and Function

Mochizuki et al. (2000) first discovered the existence of the PRDM16 gene in patients with myelodysplastic syndrome and acute myeloid leukemia, which was also named MDS1/EVI1-like gene 1 (MEL1) because of its high homology with the MDS1/EVI1 gene (Mochizuki et al., 2000). The human PRDM16 gene, located on chromosome 11p36.32, contains 17 exons and encodes a zinc finger protein with a positive regulatory (PR) domain, which also contains a proline rich domain (PRR), inhibitory domain (RD), and acidic domain (AD) (Chi and Cohen, 2016). Similarly, in mice, PRDM16 sits on the 4qE2 chromosome and contains 17 exons (Chi and Cohen, 2016). The domain also includes a PRR, an RD, and a C-terminal AD (Nishikata et al., 2003). There are four subtypes of human PRDM16 (1276 bp (full length), 1257 bp, 1276 bp, and 1092 bp (short)), of which the full length of PRDM16/MEL1 (1276 bp) and the short PRDM16/MEL1S (1092 bp) are the most widely studied subtypes (Mochizuki et al., 2000; Nishikata et al., 2003). In contrast, there are only three subtypes of mouse PRDM16 (1276 bp (full length), 1178 bp, and 1276 bp) (Chi and Cohen, 2016), and the full length of PRDM16 is known to consist of one n-terminal PR domain and two PR domains, including a C2H2 zinc finger cluster (zinc finger 1 (ZF1), which has seven zinc fingers in the N-terminus, and ZF2 has three zinc fingers in the C-terminus) (Nishikata et al., 2003). Whether mouse PRDM16 has a similar classification to short PRDM16/MEL1S needs to be further studied. PRDM16 belongs to a member of the PRDM family, which shares a PR (PRD1, BFI, and RIZ homology) domain and a zinc finger structure (Mochizuki et al., 2000). At the N-terminus, the PR structure contains approximately 100 amino acids, which have protein–protein binding sites, and at the C-terminus, the zinc finger structure has a repetitive and unequal arrangement, acting as a transcription factor (Sorrentino et al., 2018). In addition, the sequence of the PR domain is 20–30% identical to that of the SET domain, and some regions are highly homologous, which suggests that the PR domain may also have histone methyltransferase (HMT) activity, similar to histone third subunit 4 lysine trimethylation (H3K4me3) activity (Brower-Toland et al., 2009). There are four forms of zinc finger protein action, namely, transcription, protein binding, RNA binding, and simultaneous action with two types of DNA, RNA, and protein molecules (Bu et al., 2021). Therefore, PRDM16 contains a zinc finger structure that binds specifically to DNA for transcriptional activity and can recognize and bind RNA and protein, which are of the C2H2 type (Di Tullio et al., 2021). PRDM16 is expressed in many tissues, such as the pancreas (Lahortiga et al., 2004; Benitez et al., 2014), kidneys (Lahortiga et al., 2004), lungs (Lahortiga et al., 2004; Fei et al., 2019), heart (Lahortiga et al., 2004), and brain (Lahortiga et al., 2004). The expression of PRDM16 in brown adipose tissue (Chi and Cohen, 2016), lungs (Fei et al., 2019), kidneys (Kundu et al., 2020), heart (Cibi et al., 2020), and brain (Su et al., 2020) in mice is similar to PRDM16 expression in human tissues. Whether these transcripts are homologous to human PRDM16 remains to be further explored (Chi and Cohen, 2016). PRDM16 is mainly involved in lipid metabolism (Seale et al., 2007; Chi and Cohen, 2016), glucose homeostasis regulation (Hasegawa et al., 2018), mitochondrial dynamics regulation (Luchsinger et al., 2016), and oxidative stress (Chuikov et al., 2010).

### Expression Regulation

**FIGURE 1:**
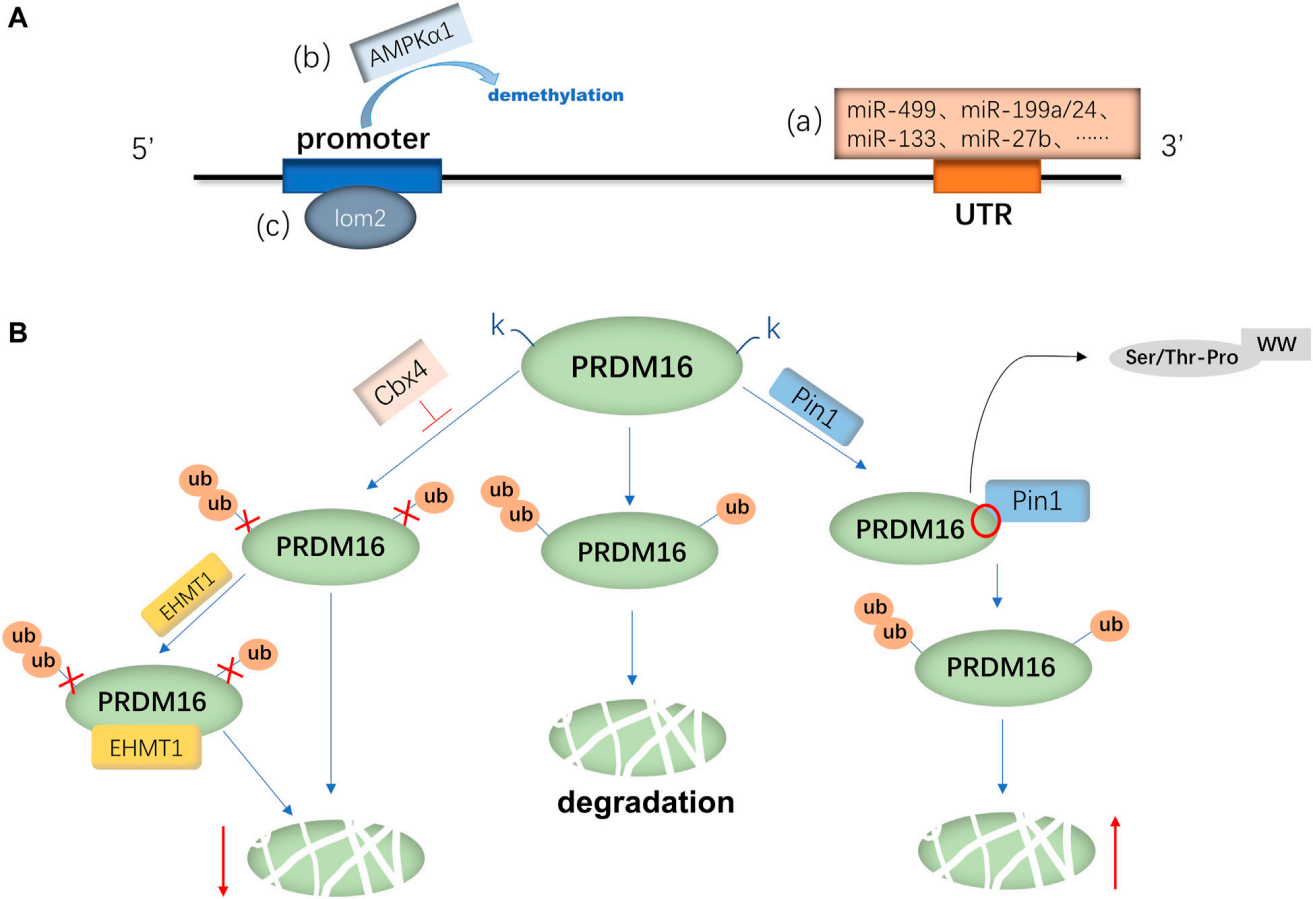
Regulatory mechanism of PRDM16 expression. **(A)** mRNA expression of PRDM16 is primarily regulated by various miRNAs(a) binding to the 3′-UTR of PRDM16 and AMPKα1(b) and lom2(c) binding to the promoter of PRDM16. **(B)** PRDM16 is degraded by ubiquitination, and Cbx4 promotes the K917 site of PRDM16, which inhibits ubiquitination; moreover, EHMT1 can further inhibit degradation. On the other hand, Pin1 facilitates the ubiquitination of the PRDM16 protein, with its WW binding to the Ser/THR-Pro site of the PRDM16 PR domain.

PRDM16 is expressed in a variety of tissues in mice and humans, and the mechanisms involved in PRDM16 expression are mainly divided into the regulation of PRDM16 transcription levels and posttranslational levels. Currently, the transcriptional regulation mechanism is widely studied (Seale et al., 2007; Chi and Cohen, 2016). First, the transcriptional regulation mechanism of PRDM16 has been studied most recently. It has been demonstrated that RNAs (miRNAs, ciRNAs, and lncRNAs) can regulate the transcription of the PRDM16 gene (Kong et al., 2015; Zhang et al., 2019; Jiao et al., 2021). For example, miR-499, miR-199a/24, miR-133, and miR-27b could negatively regulate the mRNA expression of PRDM16 by directly targeting the 3′-noncoding region (3′-UTR) of PRDM16 (Jiang et al., 2018; He et al., 2018; Trajkovski et al., 2012; Kong et al., 2015). Another study suggested that miR-448 and miR-149-3p can also inhibit PRDM16 mRNA expression (Liu et al., 2020a; Ding et al., 2016), but whether they can act on the 3′-UTR remains to be proven. In addition, the interaction between ciRNA and miRNA is known to be one of the main mechanisms regulating gene expression (Liu et al., 2019). Zhang et al. (2019) found that ciRS-133 delivered by exosomes (the prototype RNA sponge of miR-133) upregulated PRDM16 expression by binding to miR-133 (Zhang et al., 2019). The long-strand noncoding RNA reprogramming regulator (lncRNA ROR) can also activate PRDM16 transcription and increase its mRNA expression (Jiao et al., 2021) (Figure 1A). In addition, some metabolism-related factors are involved in regulating the transcriptional activity of PRDM16, such as AMP-dependent protein kinase α1 (AMPK-α1) and α-ketoglutaric acid (α-KG) (Yang et al., 2016; Peng et al., 2021). Yang et al. (2016) found that AMPK-α1 positively regulated promoter demethylation and increased the transcription of PRDM16 (Yang et al., 2016). Furthermore, α-KG is necessary for the demethylation of the PRDM16 promoter (Peng et al., 2021). Yang et al. (2016) further demonstrated that AMPK-α1 upregulates α-KG to induce PRDM16 transcriptional activity and expression (Yang et al., 2016). Furthermore, a Houston study demonstrated that T lymphocyte translocation protein 2 (LMO2) directly binds the PRDM16 promoter to promote the transcription of PRDM16 (Matrone et al., 2021). On the other hand, proteolysis is also one of the pathways that affects protein expression (Dikic, 2017), as ubiquitination or acetylation can also affect the regulation of PRDM16 expression (Chen Q. et al., 2018a; Ohno et al., 2013; Nakatsu et al., 2019; Baskaran et al., 2016). Polycomponin 4 (Cbx4) is a ubiquitin-E3 ligase (Van Wijnen et al., 2021) that promotes the ubiquitin-like reaction at the lysine 917 (K917) site of PRDM16, inhibits ubiquitination degradation of PRDM16 and enhances adipose thermogenesis (Chen Q. et al., 2018a). Euchromatin histone methyltransferase 1 (EHMT1) is an essential brown adipose tissue (BAT)-enriched lysine methyltransferase (Nachiyappan et al., 2021; Gulyaeva et al., 2019). Chen Q. et al. (2018a) found that Cbx4 promotes the ubiquitination of PRDM16, and the binding of EHMT1 to PRDM16 can further block the ubiquitination of other lysine residues and suppress the degradation of the PRDM16 protein (Chen Q. et al., 2018a; Ohno et al., 2013). In addition, Nakatsu et al. (2019) found that the WW domain of peptidyl prolyl isomerase (Pin1) can bind to the Ser/THR-Pro site of the PRDM16 PR domain, thereby promoting ubiquitination and increasing the degradation of the PRDM16 protein (Nakatsu et al., 2019) (Figure 1B). Silencing regulatory protein 1 (SITR-1) upregulates PRDM16 expression by promoting deacetylation of PRDM16 (Baskaran et al., 2016) or stabilizes the PRDM16 protein structure by promoting PPARγ deacetylation and enhancing the binding between PPARγ and PRDM16, which can increase the expression of PRDM16 (Qiang et al., 2012; Baskaran et al., 2017).

### Biological Function and Related Signaling Pathways

#### PRDM16 as a Transcription Factor

**FIGURE 2:**
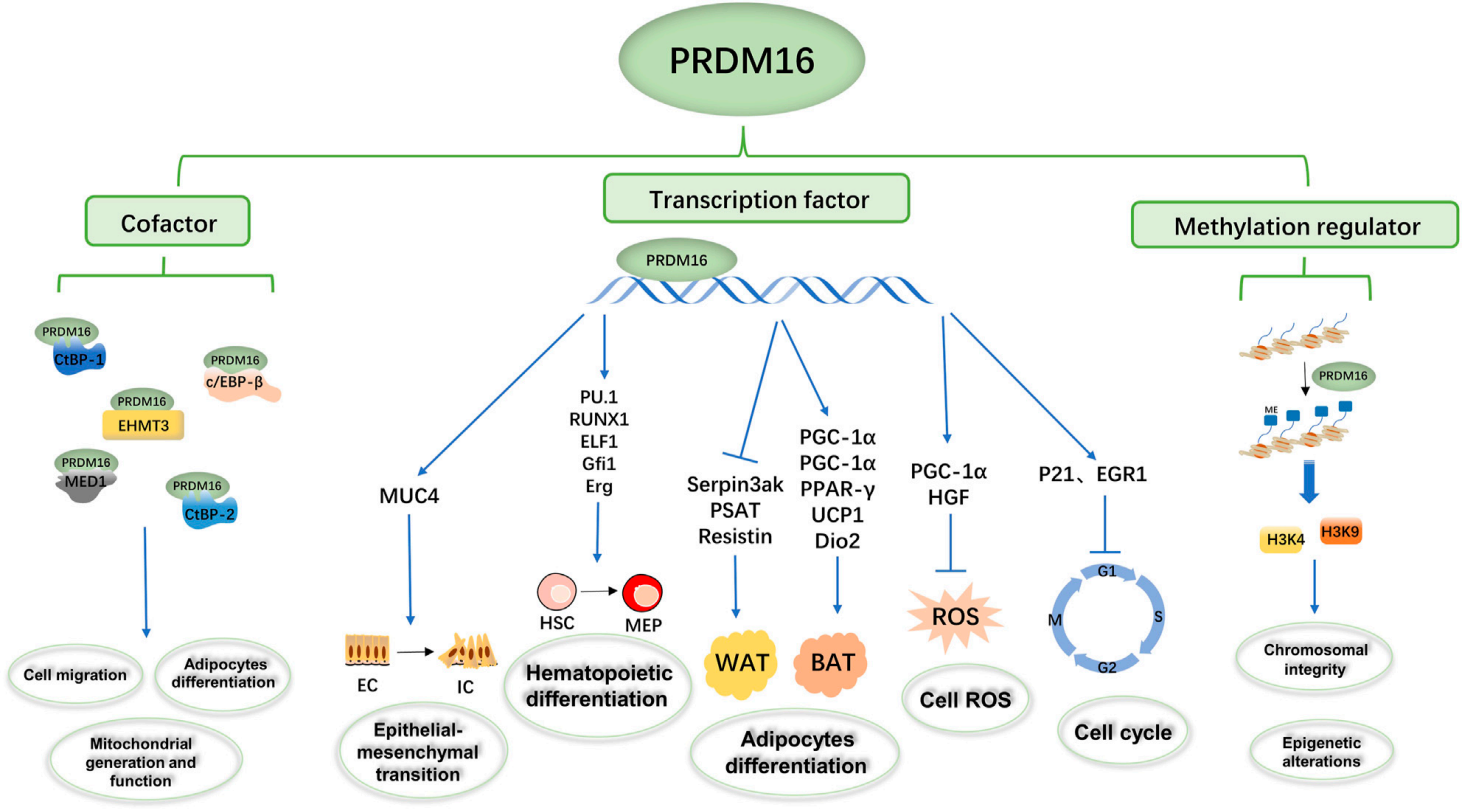
Summary of the biological functions of PRDM16. The PRDM16 PR domain participates in biological metabolism as a transcription factor, cofactor, and methylation regulator. PRDM16 could mediate epithelial–mesenchymal transformation by regulating MUC4 transcription, hematopoietic differentiation by regulating PU1, Runx1, ELF1, Gfi1, and ERG transcription, adipocyte differentiation by regulating PGC-1α, PGC-1β, PPAR-γ, UCP1, Dio2, Serpin3ak, PSAT1, and resistin transcription, and cell ROS and the cell cycle by regulating PGC-1α, HGF, P21, and EGFR transcription. In addition, PRDM16 could bind with c/EBP-β, CTBP-1, CTBP-2, EHMT3, and MED1 as transcription complexes involved in cell migration, adipocyte differentiation, and mitochondrial generation and function. Moreover, PRDM16 could mediate the methylation of H3K9 and H3K4 and then influence chromosomal integrity and epigenetic alterations.

PRDM16 is a transcription factor that can regulate the transcription of multiple genes to participate in biological metabolic processes (Figure 2). In a hematopoietic stem cell (HSC) study, Gudmundsson et al. (2020) proposed that PRDM16 directly targets cyclin-dependent kinase inhibitor 1α (CKDN1α/P21) and early growth response factor 1 (EGR1) to regulate the HSC cell cycle and induce HSC quiescence (Gudmundsson et al., 2020). In addition, Inoue et al. (2017) found that PRDM16 directly targets and promotes the expression of peroxisome receptor γ coactivator 1α (PGC1-α), thus inhibiting the production of mitochondrial reactive oxygen species (mtROS) and regulating ROS levels in cells (Inoue et al., 2017). Chuikov et al. (2010) found that PRDM16 regulates neural stem cell/progenitor cell function and ROS levels by binding to the hepatocyte growth factor/scatter factor (HGF) promoter and promoting HGF gene transcription (Chuikov et al., 2010). Several experiments have shown that PRDM16 induces the expression of brown fat-related genes, such as PGC 1α (Seale et al., 2007; Chen et al., 2018b), PGC-1β (Seale et al., 2007), PPARγ (Seale et al., 2008), uncoupling protein 1 (UCP1) (Seale et al., 2007; Iida et al., 2015), and type 2 deiodinase (Dio2) (Seale et al., 2007). On the other hand, PRDM16 inhibited the expression of white fat-related genes at the mRNA level, such as serine peptidase inhibitor 3ak (Serpin3ak), phosphoserine aminotransferase 1 (PSAT1), and resistin (Seale et al., 2007). Moreover, the overexpression of full-length PRDM16 or PRDM16-PRD inhibits transcription of mucin 4 (MUC4), which has been shown to promote epithelial–mesenchymal transformation in lung adenocarcinoma (Fei et al., 2019). In human and mouse experiments, Hu et al. (2019) found that PRDM16s activates a regulatory network of bone marrow genes organized by various bone marrow transcription factors, such as PU1, C/EBP, Runx1, and ELF1, in megakaryocyte–erythroid progenitor cells (MEPs) (Hu et al., 2019). Additionally, PRDM16 induces a variety of transcription factors (such as GFI1, Meis1, and Erg) involved in the bone marrow master regulator and stem cell biological processes to be layered upstream of the bone marrow and stem cell processes, thus giving it the ability to generate self-renewing malignant bone marrow cells (Shing et al., 2007; Hu et al., 2019).

#### PRDM16 as a Cofactor

In addition, PRDM16 directly binds the promoter to regulate transcription, and the formation of transcription complexes is also important to regulate transcription function (Chi and Cohen, 2016; Hasegawa et al., 2018; Iida et al., 2015; Kajimura et al., 2008; Ohno et al., 2013) (Figure 2). In human embryonic kidney cell 293 (HEK293) , PRDM16 and CCAAT enhancer binding protein β (c/EBP-β) bind to each other through the zinc finger structure, activate gene expression in brown fat cells, and then control the levels and function of brown fat in the body (Seale et al., 2007; Kajimura et al., 2009). C-terminal binding protein 1 (CTBP-1) and C-terminal binding protein 2 (CTBP-2) also form transcription complexes with PRDM16 (Nishikata et al., 2011), which inhibits the expression of several white adipose tissue-selective genes in a CtBP-dependent manner, activating selective gene expression in brown adipose tissue responsible for both mitochondrial biosynthesis and oxidative metabolism (Kajimura et al., 2008). Kundu et al. (2020) found that the physical binding of PRDM16 to CTBP1/2 is also necessary to suppress semaphorin 5B (SEMA5B) expression and tumor growth *in vivo* (Kundu et al., 2020). Interestingly, Kajimura et al. (2008) found that although PGC-1 and CtBP have different binding domains to PRDM16, PGC-1α and CtBP can competitively bind to PRDM16 and that the PRDM16/PGC-1α complex strongly activates the brown fat gene (Kajimura et al., 2008). Transcriptional mediator subunit 1 (MED1) is one of the components of the mediator complex (Bellacosa, 2001; Napoli et al., 2019), and PRDM16 interacts directly with the MED1 subunit of the mediator complex through the N-terminal region of its zinc finger structure, which is recruited into the superenhancer of the brown fat selection gene (Harms et al., 2015). In addition, PRDM16 could be recruited into the enhancer of the brown fat-specific uncoupling protein 1 (UCP1) gene through this interaction and in a media-dependent manner in enhanced thyroid hormone receptor (TR)-driven transcription (Iida et al., 2015). In cardiomyocytes, PRDM16 inhibits the expression of Myc oncogene homolog (Myc), a transcription factor-promoting hypertrophy, by synergistic action with EHMETs and reduces pathological myocardial hypertrophy (Cibi et al., 2020).

#### PRDM16 Regulates Gene Methylation

DNA methylation is one of the common epigenetic mechanisms that inhibit gene transcription (Unnikrishnan et al., 2019), involving mechanisms such as DNA conformation that directly inhibit the binding of DNA to transcription factors and to methylated DNA-binding proteins (such as methylcpG binding protein 2, MeCP2) to form transcription inhibitory complexes (Brower-Toland et al., 2009; Greenberg and Bourc'his, 2019). PRDM16 is a nuclear membrane protein and a highly specific histone H3K4 methyltransferase on chromatin that participates in biological metabolism (Biferali et al., 2021; Zhou et al., 2016; Pinheiro et al., 2012) (Figure 2). PRDM16, located in the NL region, works with the H3K9 methyltransferase G9a/GLP to mediate the silencing of myogenic genes; thus inhibiting another myogenic pathway of fibroadipogenic progenitors (FAPs) (Biferali et al., 2021). In mouse embryonic fibroblasts, PRDM16 directly mediates H3K9me1 site methylation and regulates major satellite transcription, which is critical for mammalian heterochromosome integrity (Pinheiro et al., 2012). The H3K4 methyltransferase activity of PRDM16 promotes the expression of transcription factor-independent growth factor 1B (GFI1B), and thus inhibits the expression of the homeobox (Hox) gene, suppressing mixed lineage leukemia (MLL) progression (Zhou et al., 2016).

#### Protein–Protein Interactions With PRDM16

The direct binding of some proteins to PRDM16 may also affect its function (Hondares et al., 2011; Lodhi et al., 2017; Hasegawa et al., 2018). It was found that the peroxisome receptor α (PPARα) protein binds directly to zinc finger 1 (ZF1) and zinc finger-2 (ZF2) of PRDM16 (Seale et al., 2008; Villanueva et al., 2013), facilitating the binding of PRDM16 and PGC1-α, which could induce the expression of genes involved in thermogenesis (Hondares et al., 2011). In addition, peroxisome lipid synthase (PexRAP) also binds to PRDM16 and peroxisome proliferator receptor R (PPARγ), and the downstream inhibition of PRDM16 is involved in browning and thermogenesis-related gene expression in adipose tissue (Lodhi et al., 2017). Furthermore, the coactivation of transcription factor H2.0 homeobox (HLX) with PRDM16 DNA could induce browning and thermogenesis of adipose tissue (Lodhi et al., 2017). Hasegawa et al. (2018) found that GTF2IRD1 inhibits obesity-related adipose tissue fibrosis in humans and mice by recruiting PRDM16 and Ehmt1 into the promoter or enhancer region of the growth suppressor β gene, improving glucose metabolism and homeostasis (Hasegawa et al., 2018).

## Adipocyte Transformation and Thermogenesis

**FIGURE 3:**
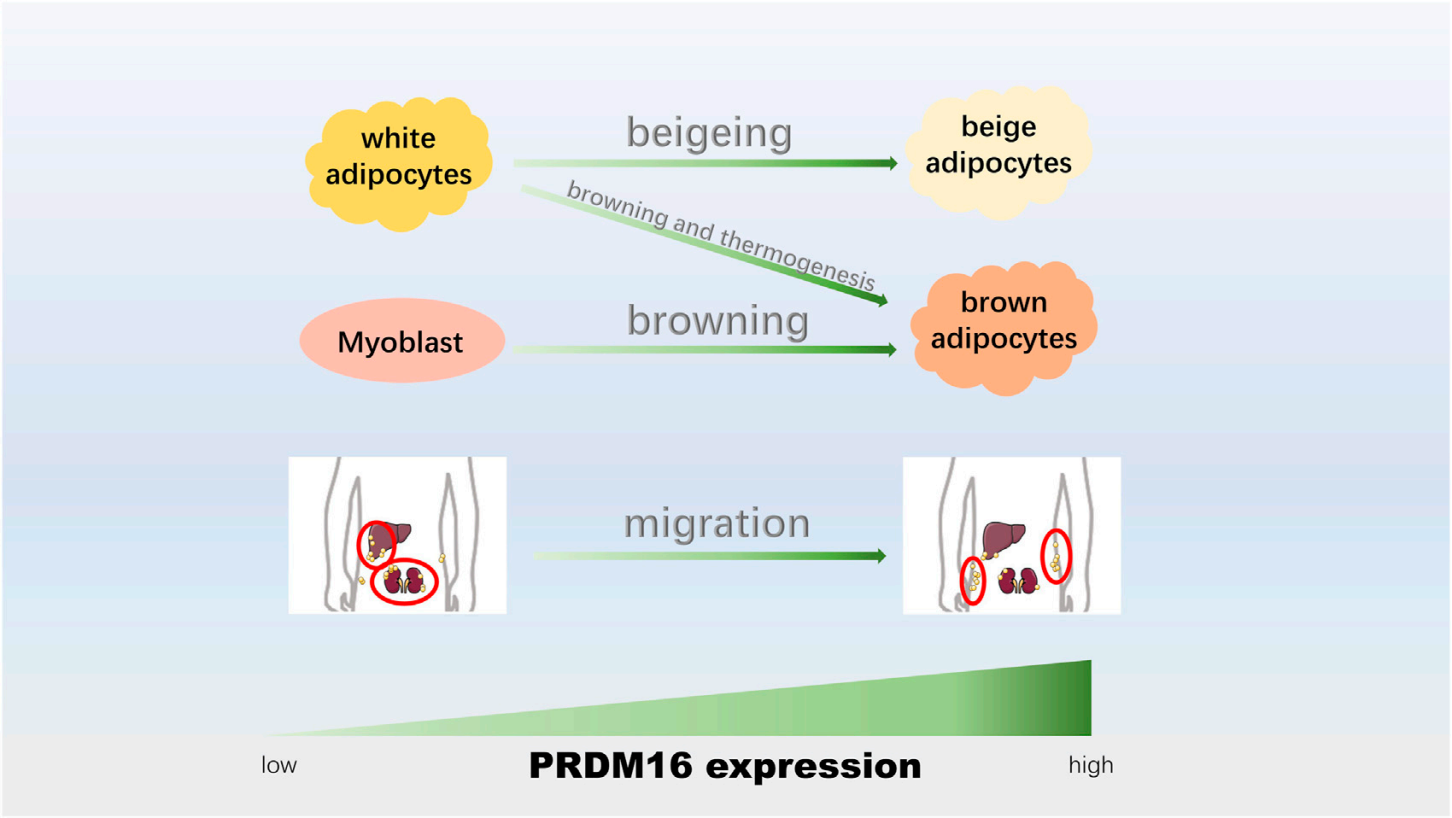
PRDM16 participates in adipocyte transformation and thermogenesis. The upregulation of PRDM16 could promote the beigeing of white adipocytes, adipose tissue browning and thermogenesis, adipogenic differentiation of myoblasts, and conversion from visceral fat to epidermal fat.

Three types of fat cells are white fat (Ying and Simmons, 2020), which is responsible for storing energy (Bielczyk-Maczynska, 2019), and brown fat and beige fat, which are responsible for productivity (Hussain et al., 2020). Under different stimuli, different adipocytes can be transformed into each other, such as the browning of white adipocytes (transforming into brown adipocytes) (Bartelt and Heeren, 2014) and beige adipocytes (transforming into beige adipocytes) (Velazquez-Villegas et al., 2018), which could enhance mitochondrial activity, improve glucose metabolism abnormalities and promote energy consumption (Harms and Seale, 2013; Bartelt and Heeren, 2014). In addition, the data showed an association between muscle cells and brown fat cells (Borensztein et al., 2012; Yin et al., 2013), and the differentiation between these two cells is of great significance for the formation and distribution of muscle in humans. The location distribution of human fat is roughly divided into subcutaneous and visceral fat (Ding et al., 2016), and the translocation of visceral fat to subcutaneous fat is beneficial to the human body (Seale et al., 2011; Ding et al., 2016). Recently, studies have shown that PRDM16 plays a critical role in adipocyte transformation and thermogenesis (Figure 3).

### PRDM16 Maintains Brown Adipose Tissue Morphology and Thermogenesis

PRDM16 is highly expressed in adipose tissue (Seale et al., 2011; Ohno et al., 2013), and PRDM16 promotes the differentiation of white fat precursors into brown fat cells (Ohno et al., 2012; Trajkovski et al., 2012). Transcriptome results showed that the PRDM16 gene was involved in the development of wild-type BAT (Chi and Lin, 2018). PPARγ ligand is known to induce browning of white adipocytes (Ohno et al., 2012), and data have shown that PRDM16 stimulates brown adipogenesis by binding to PPARγ and activating its transcriptional function (Seale et al., 2008; Qiang et al., 2012). The absence of PRDM16 attenuates the effects of rosiglitazone (a PPARγ agonist) on the brown fat gene program, suggesting that PRDM16 expression is required for full activation of PPARγ ligands to preferentially induce brown fat gene expression in subcutaneous white fat (Ohno et al., 2012). By constructing fat-specific Cbx4 knockdown mice, Chen Q. et al. (2018a) found that Cbx4 had a significant effect on white fat remodeling and was proven to regulate white fat browning by controlling the stability of PRDM16 (Chen Q. et al., 2018a). Gan et al. (2018) proposed that Foxc2 ameliorates inflammation and promotes fat browning in high-fat diet (HFD)-induced obese mice by reducing the leptin-mediated JAK2/STAT3/PRDM16 pathway (Gan et al., 2018). In addition, Zhang et al. (2019) found that ciRS-133 knockout reduced the occurrence of cachexia, increased the expression of PRDM16 in tumor tissues, and decreased oxygen consumption and heat production (Zhang et al., 2019). In addition to affecting the formation of brown fat, PRDM16 is closely related to the burning of brown fat.

Nonshivering thermogenesis of adipocytes has a protective effect on hypothermia and obesity (Li et al., 2021). Loss of Pin1 in differentiated adipocytes enhanced the thermogenesis of the β3 agonist CL316243, possibly through the upregulation of PRDM16 protein (Nakatsu et al., 2019). *In vivo* data showed that PRDM16 deficiency significantly reduced the thermogenic characteristics of BAT interscapular in adult mice (Harms et al., 2014). PRDM16-overexpressing mice presented increased energy expenditure, limited weight gain, and improved glucose tolerance and responded to a high-fat diet (Seale et al., 2011). The complex of PPARα, PGC-1α, and PRDM16 regulates the expression of lipid catabasis and thermogenic genes and is a key component of heat production in brown fat (Hondares et al., 2011). In addition, the overexpression of the PRDM16 PR region inhibited the differentiation of preadipocytes and significantly increased lipolysis levels and mitochondrial oxidation capacity during differentiation (Gu et al., 2019). PRDM16 is a key coregulatory protein required for the “browning” of white fat (Ding et al., 2016) and is a cellular autonomic determinant of brown adipose-like gene programming and thermogenesis in subcutaneous adipose tissue (Seale et al., 2011).

### PRDM16 Promotes the Adipogenic Differentiation of Myoblasts

Brown and white fat cells were previously thought to be derived from the same precursor cell, despite histological and functional differences (Frühbeck et al., 2009). Experiments have shown that myoblasts and adipocytes are derived from a common mesodermal precursor, suggesting a close correlation between muscle and adipocytes (Borensztein et al., 2012). Lineage tracing revealed that brown fat cells were derived from myogenic progenitor cells during embryonic development (Yin et al., 2013). *In vivo* experiments showed that brown fat cells were generated from the Myf5(+) myoblast lineage by PRDM16 (Seale et al., 2008). Borensztein et al. (2012) found that mutated mice with defective IGF2 and MyoD genes showed extensive BAT hyperplasia, and increased adipocyte proliferation in BAT mice with defective IGF2 and MyoD genes was associated with the overexpression of brown fat-specific marker uncoupled protein 1 (UCP1) (Borensztein et al., 2012). The expression of PRDM16, a major key gene involved in the transition between myogenic and brown adipogenic lineages, was significantly increased (Seale et al., 2007; Borensztein et al., 2012). MiR-133 regulates myogenic and brown fat assay selection by targeting PRDM16’s 3′UTR and affects muscle regeneration (Yin et al., 2013). MiR-499 inhibits adipogenic differentiation of skeletal muscle satellite cells (SMSCS) by negatively regulating the expression of PRDM16, and the overexpression of PRDM16 partially reversed this effect (SMSCS) (Jiang et al., 2018). Therefore, PRDM16 is a key factor that induces the differentiation of skeletal muscle precursors into brown adipocytes and inhibits myogenic differentiation (Seale et al., 2008; Jiang et al., 2018). PRDM16-induced transdifferentiation of mouse myoblasts (C2C12) is associated with changes in MyoD CpG methylation, which affects myogenesis and adipogenesis by regulating histone methylation markers on MyoD and PPARγ promotors (Li et al., 2015). Kajimura et al. (2009) found that PRDM16 forms a transcription complex with the active form of C/EBP-beta (also known as LAP) that controls cell conversion from myoblast precursors to brown fat cells. The PRDM16-C/EBP-β complex initiates brown fat formation of the myoblast precursor (Kajimura et al., 2009). Deficiency of PRDM16 in brown fat precursors results in the loss of brown fat characteristics and promotes muscle differentiation, and in brown fat, it leads to morphological abnormalities, decreased expression of thermogenic genes, and increased expression of muscle-specific genes (Seale et al., 2008). Frühbeck et al. (2009) proposed that the overexpression of PRDM16 determines the development of brown fat cells from progenitor cells-expressing myoblast markers, while loss of PRDM16 from these precursors does not lead to differentiation of white fat cells (Frühbeck et al., 2009). In conclusion, PRDM16 controls the adipogenic differentiation of myoblasts and plays an important role in regulating the balance of adipose tissue and muscle tissue (Seale et al., 2008; Frühbeck et al., 2009; Kajimura et al., 2009).

### PRDM16 Promotes Epidermal and Visceral Fat Conversion

White fat organs are composed of subcutaneous and intraperitoneal fat pools, and abdominal obesity is a major risk factor for metabolic diseases in rodents and humans, while subcutaneous fat is relatively benign (Seale et al., 2011; Ding et al., 2016). After a 24-h fast, the subcutaneous adipose tissue of mice acquired key properties of visceral fat, and the mechanism involved may be that mir-149-3p directly upregulates PRDM16 expression (Ding et al., 2016). Obese animals on a high-fat diet were associated with severe insulin resistance and hepatic steatosis, and their fat distribution also changed; that is, subcutaneous fat increased significantly (Airaksinen et al., 2018). The subcutaneous adipose tissue of PRDM16 mutant mice acquired many key properties of visceral fat, including reduced thermogenicity, increased expression of inflammatory genes, and increased macrophage aggregation (Cohen et al., 2014). In a clinical study, with 33 overweight/obese and 28 normal-weight individuals, Liu L. et al. (2021a) suggested that PRDM16 promoter methylation levels were higher in overweight/obese individuals than in healthy control individuals (Liu L. et al., 2021a). Furthermore, they analyzed the methylation levels of the PRDM16 promoter in abdominal subcutaneous fat (SAT) and omental adipose tissue (OAT) and found that seven CpG methylation levels of the PRDM16 gene in abdominal OAT were increased compared with those in SAT (Liu L. et al., 2021a). These results suggest a complex relationship between PRDM16 gene methylation levels and overweight/obesity, which may affect the distribution of adipocytes in the body (Serrano et al., 2020; Liu L. et al., 2021a).

### PRDM16 Promotes the “Beigeing” of White Fat

As mentioned earlier, in addition to brown fat producing heat, beige fat also produces heat and thus has metabolic benefits (Rabiee, 2020). The fat cells of PRDM16 knockout mice not only lost the thermogenic properties of beige fat cells but also acquired some harmful properties, including the accumulation of macrophages (Cohen et al., 2014). Cohen et al. (2014) found that after cold exposure or β3 agonist treatment, the loss of PRDM16 had little effect on classical brown fat but significantly inhibited the function of beige fat cells in subcutaneous fat (Cohen et al., 2014), suggesting that PRDM16 plays a thermogenic role mainly by regulating beige fat in some special cases (Auffret et al., 2012; Cohen et al., 2014). However, precursors of metabolically beneficial beige adipocytes may also become fibrogenic and promote lipofibrosis (Wang et al., 2019; Maharjan et al., 2021). Increased PRDM16 was found to reduce fibrosis and restore beige adipogenesis in aging mice, and the possible mechanism is that PRDM16 inhibits precursor fiber formation and enhances beige adipogenesis by regulating the secretion of the metabolite β-hydroxybutyrate (BHB) by adipocytes (Wang et al., 2019). In addition, the PRDM16 transcription complex was found to effectively inhibit adipose tissue fibrosis in a UCP1-independent manner (Hasegawa et al., 2018). Hasegawa et al. (2018) proposed that the PRDM16 transcription complex also powerfully inhibits adipose tissue fibrosis through direct interaction with GTF2IRD1 to protect animals from diet-related glucose tolerance and *in vivo* insulin resistance (Hasegawa et al., 2018).

In addition to the aforementioned effects, analysis of the brown fat lineage showed that PRDM16 was indispensable in the development of BAT embryos, suggesting that PRDM16 controls the characteristics and functions of BAT after birth (Harms et al., 2014). Park et al. (2019) constructed the adipose-specific knockout peroxisome biogenic factor Pex16 (Pex16-AKO) mice that were genetically modified to reduce cold tolerance and energy expenditure and to aggravate diet-induced obesity. Further research revealed that Pex16 protects against cold exposure by activating the thermoregulator PRDM16 (Park et al., 2019). PRDM16 regulates fat cell metabolism through multiple mechanisms and controls the fate of fat cells (Seale et al., 2007; Seale et al., 2008; Cohen et al., 2014; Park et al., 2019; Liu L. et al., 2021a), which may be of great significance for metabolic diseases closely related to fat cells, such as obesity and diabetes (Chondronikola et al., 2016; Shao et al., 2016; Shankar et al., 2019).

## PRDM16 in Obesity and Diabetes

### Evidence of PRDM16 in Obesity and Diabetes

Brown adipose tissue is abundant in newborns, but almost absent in adults (Cypess et al., 2009). Since BAT can generate heat and consume energy, promoting the development of brown adipose tissue is a promising strategy for combating obesity and related metabolic disorders (Yang et al., 2016). Genetic modification of mice revealed that PRDM16 is critical for pancreatic development, suggesting that PRDM16 may be a regulatory gene for pancreatic development and related diseases such as diabetes (Benitez et al., 2014). In addition, Côté et al. (2016) found that the children of pregnant women with gestational diabetes mellitus (GDM) had a higher risk of obesity and T2DM, possibly associated with BAT. The changes in the DNA methylation levels of PRDM16 and PGC-1α were associated with blood leptin levels in the umbilical cord after fetal exposure to maternal hyperglycemia (Côté et al., 2016). It is well-known that both PRDM16 and PDE4 genes are the essential regulators of heat production in BAT (Alamrani et al., 2018). A Saudi population study showed that the PRDM16 polymorphism (RS2651899) is a risk factor for obesity and significantly affects blood lipids. However, the PDE4D (RS295978) polymorphism did not show a significant influence on the risk of obesity or the lipid profile (Alamrani et al., 2018). In addition, epigenetic variation may be an important factor in the development of complex metabolic diseases such as type 2 diabetes mellitus (T2DM) (Ling and Rönn, 2019). To investigate the genome-wide DNA methylation pattern in the liver of T2DM and nondiabetic controls and the epigenetic changes related to gene expression, Nilsson et al. (2015) used a human methylation 450k BeadChip (HumanMethylation450 BeadChip). Compared with nondiabetic subjects, they found that 251 CpG sites in the liver obtained by T2DM showed different DNA methylation, including the PRDM16 gene (Nilsson et al., 2015), suggesting that PRDM16 gene methylation is closely related to T2DM. Data from a full epigenome association study showed that CpG hypermethylation near PRDM16 in the offspring of mothers with T2DM during pregnancy (OMD) also predicted future diabetes risk, impacting insulin secretion, increased body weight, and increased risk of developing T2DM (Chen et al., 2017). These data suggest that PRDM16 is closely related to blood lipids, blood glucose, pancreatic development, and even to obesity and T2DM.

### The PRDM16 Signaling Pathway May Ameliorate the Pathogenesis of Obesity and Diabetes

It was found that obesity, insulin resistance, and cirrhosis were induced by a high-fat diet in mice, and pharmacologically activated PRDM16 effectively alleviated the symptoms of HFD (Cohen et al., 2014). Long-term GEN treatment reduces white adipose tissue (WAT) inflammation and liver adipogenesis, promotes WAT brown induction (Shen et al., 2019), and prevents HFD-induced weight gain by regulating the AMPK/PRDM16/UCP1 pathway (Choi et al., 2017). Intraperitoneal injection of L-theanine (100 mg/kg/day) upregulated the expression of PRDM16, UCP1, and other thermogenic genes, enhanced the adaptive thermogenic effect and induced the browning of white adipose tissue in the groin (iWAT), and played a role in nondiet-related obesity in mice (Peng et al., 2021). In addition, activation of TRPV1 in BAT enhanced SIRT 1 expression and promoted the deacetylation and interaction of PPARγ and PRDM16 (Baskaran et al., 2017). Dietary addition of capsaicin promoted WAT browning to fight obesity but did not prevent obesity in TRPV1^−/−^ mice, suggesting that activation of the TRPV1 pathway promotes the interaction between PPARγ and PRDM16 protein to protect against obesity (Baskaran et al., 2016). In addition, Imran et al. (2017) found that cryptotanshinone (CT) could upregulate PRDM16, PGC1-α, and UCP1 protein expression; downregulate the white fat marker resistin protein expression; and activate oxidative phosphorylation and the AMPK signaling pathway, thereby reducing the fat content in mouse embryonic fibroblasts (3T3-L1 cells) and mesenchymal stem cells (C3H10T1/2 cells), implying that the PRDM16 pathway may be involved in the promising antiobesity effect of CT (Imran et al. 2017). Zheng et al. (2019) provided direct evidence for the antiobesity and antidiabetes effects of PRDM16 for the first time and identified the mir-149-3p/PRDM16 signaling pathway as a target for the prevention and treatment of obesity and metabolic dysfunction (Zheng et al., 2019). Pomegranate seed oil (PSO) enhances thermogenic genes, mitochondrial signaling, and lipid metabolism by increasing the expression of Mfn2, OPA1, PRDM16, and PGC-1α and increasing insulin receptor phosphorylation and thermogenic genes. It reduces obesity-mediated insulin resistance and the progression of liver fibrosis and has potential therapeutic effects in the prevention of obesity-related metabolic disorders (Raffaele et al., 2020). PRDM16-related signaling pathways are involved in the pathogenesis of obesity and diabetes, and intervening in PRDM16 expression or affecting the function of PRDM16 is likely to be an effective strategy to treat these diseases.

### PRDM16 May Serve as a Potential Therapeutic Target in Obesity and Diabetes Treatment

In obese and diabetic patients, a study showed that the targeted cellular approach of introducing the PRDM16 gene into embryos to induce a brown fat phenotype may be an effective strategy for the treatment of metabolic diseases (Kishida et al., 2015). Studies have shown that PRDM16 and its related coregulatory factors PGC-1α and CTBP1/2 are potential targets for obesity-related therapy, controlling WAT-to-BAT conversion (Farmer, 2008). It is of concern that some drugs for obesity or diabetes affect brown fat and thermogenesis, possibly through the activation of the PRDM16 signaling pathway (Table 1). For example, acadesine (AICAR) or metformin are recognized drugs for the treatment of obesity and diabetes (Towler and Hardie, 2007). Yang et al., 2016 found that the activation of AMPK by AICAR or metformin can rescue obesity-induced brown fat production and thermogenesis inhibition, which may be related to the AMPK-α1/αKG/PRDM16 signaling pathway (Yang et al., 2016). Ohno et al. (2012) proposed that rosiglitazone activates the brown fat gene program *in vivo*, promoting heat production and weight loss *via* the PPARγ/PRDM16 pathway (Ohno et al., 2012). Zhou et al. (2019) found that liraglupeptide can induce the differentiation of brown adipocytes in skeletal muscle, including the expression of UCP1 and PRDM16 proteins, and improve insulin sensitivity through multiple pathways, thereby reducing inflammation, enhancing fatty acid oxidation, and inducing adaptive thermogenesis (Zhou et al., 2019). Compound C (an AMPK inhibitor) downregulated PRDM16 in adipocytes, and the effect of rutaecarpine on adipocyte browning was eliminated (Liu et al., 2021b). Liu X. et al. (2021b) proposed that rutaecarpine may play a therapeutic role in obesity using AMPK/PRDM16 signaling (Liu et al. 2021b). Pan et al. (2019) found that resveratrol, as a natural stilbene with antiobesity effects, can enhance the expression of thermogenic-related proteins UCP1 and PRDM16 by activating SIRT1/PGC-1α (Pan et al., 2019). Mulberry leaves have been proven to have a variety of biological activities, such as antidiabetic and anti-inflammatory effects (Chen et al., 2022). Cheng et al. (2022) showed that mulberry may induce the browning of iWAT by increasing the expression of brown marker genes and beige-specific genes (such as PRDM16) (Cheng et al., 2022). Multiple experimental data suggest that stimulating or stabilizing PRDM16 expression and inducing PRDM16 function may be a potential way to treat obesity and diabetes.

**TABLE 1:** PRDM16 signaling may have antiobesity and antidiabetes effects.

Object of study	Drugs	PRDM16 expression	Potential pathway	Results	Ref
Prkaa1^−/−^ mice	AICAR or metformin	Upregulation	AMPKα1/α-KG pathway activates PRDM16 DNA demethylation *via* TET mediation	Impairing BAT development and glucose tolerance and decreasing visceral fat weight	[54]
C57BL/6J mice	Rosiglitazone	Upregulation	PPARγ ligands stabilize PRDM16 *via* ubiquitin–proteasome pathway	Activating the thermogenic brown fat gene program and BAT development	[103]
C57BL/6J mice	Liraglupeptide	Upregulation	Inducing the expression of PRDM16	Improving glucose tolerance and insulin sensitivity and losing weight	[139]
Ucp1-luciferase mice	Rutaecarpine	Upregulation	Activating the AMPK/PRDM16 axis	Promoting adipocyte browning and reducing food intake	[140]
C57BL/6J mice	Resveratrol	Upregulation	Sirt1 or PPARα activates PRDM16 expression *via* PGC-1α signaling	Inducing thermogenesis in beige adipose tissues and reducing body weight and food intake	[141]
Sprague–Dawley rats	Mulberry	Upregulation	AMPK/PGC-1α pathway activates PRDM16 expression	Ameliorating glucose tolerance and insulin sensitivity, inducing browning, and reducing body weight	[143]

In addition to its anti-disease effects by promoting brown fat browning and brown fat thermogenesis, PRDM16 also protects against adverse outcomes by affecting lipofibrosis and glucose homeostasis (Yin et al., 2013; Huang et al., 2017; Wang et al., 2019). Adipose tissue fibrosis is becoming a marker of unhealthy adipose tissue (Hasegawa et al., 2018). PRDM16 protects against aging and fatty fibrosis caused by a high-fat diet (Wang et al., 2019). A study showed that Hlx gene expression at the physiological level drives a complete thermogenesis process through PRDM16 coactivation and converts white fat to brown-like fat, thereby improving glucose homeostasis and preventing obesity and hepatic steatosis (Huang et al., 2017). In addition, antagonistic miR-133 also improves glucose tolerance by targeting PRDM16, increasing uncoupled respiration, glucose uptake, and thermogenesis (Yin et al., 2013).

## Conclusion

In summary, PRDM16 plays a crucial role in adipocyte transformation and thermogenesis by inducing the transformation of white adipocytes or muscle cells into brown adipocytes, the transformation of white adipocytes into beige adipocytes, the thermogenesis of adipocytes, and the migration of visceral adipocytes subcutaneously. Browning and thermogenesis of brown fat cells are dominant. As a transcription factor, PRDM16 protein has multiple functions and plays an important role in physiological and pathological processes. A number of studies have shown that PRDM16 gene polymorphisms or methylation is associated with lipid levels or insulin development. Hypermethylation of PRDM16 increases the risk of obesity and diabetes, suggesting that PRDM16 may be a very effective therapeutic target for obesity and diabetes. In addition, some well-known drugs alleviate obesity and diabetes by regulating PRDM16-related signaling pathways. In conclusion, PRDM16 plays an important role in adipocyte transformation and thermogenesis, which are closely related to the occurrence of obesity and diabetes. This evidence suggests that the PRDM16 protein may be a promising therapeutic target for obesity and diabetes.
